# Enforced expression of miR-92b blunts *E. coli* lipopolysaccharide-mediated inflammatory injury by activating the PI3K/AKT/β-catenin pathway via targeting PTEN

**DOI:** 10.7150/ijbs.56933

**Published:** 2021-03-25

**Authors:** Kangfeng Jiang, Jing Yang, Chunlian Song, Fengping He, Liangyu Yang, Xiaobing Li

**Affiliations:** 1 College of Veterinary Medicine, Yunnan Agricultural University, Kunming 650201, Yunnan, People's Republic of China.; 2 State Key Laboratory of Agricultural Microbiology, College of Veterinary Medicine, Huazhong Agricultural University, Wuhan 430070, People's Republic of China.

**Keywords:** endometritis, miR-92b, inflammation, apoptosis, PI3K/AKT

## Abstract

Endometritis is a reproductive disorder characterized by an inflammatory response in the endometrium, which causes significant economic losses to the dairy farming industry. MicroRNAs (miRNAs) are implicated in the inflammatory response and immune regulation following infection by pathogenic bacteria. Recent miRNA microarray analysis showed an altered expression of miR-92b in cows with endometritis. In the present study, we set out to investigate the regulatory mechanism of miR-92b in endometritis. Here, qPCR results first validated that miR-92b was down-regulated during endometritis. And then, bovine endometrial epithelial cells (BEND cells) stimulated by high concentration of lipopolysaccharide (LPS) were employed as an *in vitro* inflammatory injury model. Our data showed that overexpression of miR-92b significantly suppressed the activation of Toll-like receptor 4 (TLR4) and nuclear factor-κB (NF‐κB) in LPS-stimulated BEND cells, thereby reducing pro-inflammatory cytokines release and inhibiting cell apoptosis. Looking into the molecular mechanisms of regulation of inflammatory injury by miR-92b, we observed that overexpression of miR-92b restrained TLR4/NF‐κB by activating the phosphatidylinositol 3-kinase/protein kinase B (PI3K/AKT)/β-catenin pathway. Furthermore, the luciferase reporter assay suggested that miR-92b targeted inhibition of phosphatase and tensin homolog (PTEN), an inhibitor of the PI3K/AKT/β-catenin pathway. Importantly, *in vivo* experiments confirmed that up-regulation of miR-92b attenuated the pathological injury in an experimental murine model of LPS-induced endometritis. Collectively, these findings show that enforced expression of miR-92b alleviates LPS-induced inflammatory injury by activating the PI3K/AKT/β-catenin pathway via targeting PTEN, suggesting a potential application for miR-92b-based therapy to treat endometritis or other inflammatory diseases.

## Introduction

Reduced reproductive performance in dairy cows is often associated with uterine diseases. Cow endometritis is a common reproductive disorder disease, which leads to huge economic losses to the dairy industry 1. Among the many factors that cause endometritis in dairy cows, infection by pathogenic microorganisms is the leading cause of endometritis 2.

After parturition, a number of external pathogenic microorganisms, particularly pathogenic *E. coli*, invade the uterine lumen and adhere to the endometrium. As the first barrier to the endometrial immune response, endometrial epithelial cells sense the presence of pathogenic bacteria through innate immune receptors, such as Toll-like receptor 4 (TLR4), which leads to the activation of downstream nuclear factor-κB (NF-κB) 3. NF-κB is a key transcription factor that can induce the release of inflammaory mediators including TNF-α and IL-6 4. And then, these inflammatory mediators recruit inflammatory cells, especially neutrophils and macrophages, to reach the site of infection, in order to eliminate invading pathogens 3. Therefore, an appropriate inflammatory response contributes to the elimination of pathogenic bacteria, but the excessive and persistent inflammation can lead to cell apoptosis and endometrial damage, which in turn causes early embryo loss and other reproductive disorders 5. Thus, to restrain the inflammatory process and alleviate tissue damage, the activation of TLR4 signal transduction should be tightly controlled.

MicroRNAs (miRNAs) are a type of single-stranded non-coding RNAs, which are typically about 22 nucleotides in length 6, 7. miRNAs can modulate gene expression through affecting the translation or stability of the target messenger RNAs (mRNAs) 8. Calin et al. reported that more than 50% of miRNA genes are located in genomic regions associated with cancer 9. It is now well established from a variety of studies that miRNAs are involved in tumor progression as oncogenes or tumor suppressors 10, 11. In addition, miRNAs also play essential regulatory roles in inflammatory and immune responses 12. By suppressing signal transduction proteins that mediate intracellular signaling, or by directly targeting mRNAs encoding specific inflammatory cytokines, miRNAs can significantly regulate the process of the inflammatory response 13. More importantly, alteration of miRNAs expression is closely associated with the development of cow endometritis, among which miR-92b displays a low expression pattern in cows with endometritis 14.

miR-92b, a highly conserved miRNA in many species, has become an attractive target for drug therapy against various cancers. miR-92b contributes to glioma proliferation and invasion by modulating Wnt/β-catenin signaling 15. However, another study showed that miR-92b is able to prevent the proliferation and invasion of lung cancer cells through targeted inhibition of EZH2 16. Importantly, miR-92b has also been reported to be a key regulator in inflammatory diseases, such as acute pancreatitis 17. Nevertheless, there are limited mechanistic studies on miR‐92b's role in the pathogenesis of endometritis. In the present study, we reported the first data describing the crucial role of miR-92b in the inflammatory responses in bovine endometrial epithelial cells (BEND cells) following LPS stimulation, as well as its therapeutic potential in a murine model of endometritis.

## Materials and Methods

### Collection of bovine uterus samples

Healthy or inflamed uterus samples (n=3) were collected according to the protocols approved by the Huazhong Agricultural University Animal Care and Use Committee (Wuhan, China) and then stored at -80 °C for subsequent analysis.

### Cell culture and treatment

BEND cells and HEK293T cells were obtained from American Type Culture Collection (ATCC). BEND cells were cultured in Dulbecco's modified Eagle's medium (DMEM)/F-12 (HyClone, USA) with 10% foetal bovine serum (FBS, Gibco, USA). HEK293T cells were grown in RPMI 1640 (HyClone, USA) with 10% FBS. To investigate the functional role of miR‐92b in the inflammatory injury, BEND cells were treated with 10 μg/mL LPS (Sigma, USA) or with other corresponding treatments. After the indicated treatments, the cells were subject to further studies.

### Cell transfection

miR‐92b mimic or inhibitor, phosphatase and tensin homolog (PTEN) siRNA (si‐PTEN) and corresponding negative controls (NC) were designed and synthesized by GenePharma (Shanghai, China). The synthesized miR‐92b mimic/inhibitor or si‐PTEN were transfected into cells at a final concentration of 100 nM by using Lipofectamine 2000 (Invitrogen, USA) following the manufacturer's instructions. Overexpression of PTEN in cells was achieved via transfection with 4 μg pcDNA3.1-PTEN plasmids.

### Plasmids construction and luciferase reporter assay

The wild-type (wt) and mutant-type (mut) luciferase reporter plasmids psiCHECK2-PTEN-3′-UTR were constructed by cloning the PTEN mRNA 3′‐UTR sequences containing the binding site into the psiCHECK2 plasmids. HEK293T cells were cotransfected with luciferase reporter plasmids and miR‐92b mimic or NC by using Lipofectamine 2000 (Invitrogen, USA) according to the manufacturer's suggestions. For luciferase reporter assay, the luciferase activities were measured by using the Dual‐Luciferase Reporter Assay System (Promega, USA) 24 h after transfection according to the manufacturer's protocol.

### Mouse Model and Sampling

Female BALB/c mice at 6-8 weeks old were provided by the Experimental Animal Center of Huazhong Agricultural University (Wuhan, China). All mice were housed in the pathogen-free animal facilities of Huazhong Agricultural University. All animal experiments in the present study were performed according to the guidelines provided by the Ethical Committee on Animal Research at Huazhong Agricultural University.

To unravel whether miR-92b acts as a potential target for the treatment of endometritis, the mice were randomly assigned into four groups (n=10): control group, LPS group, agomir-92b + LPS group and agomir-NC + LPS group. A murine model of LPS-induced endometritis was established as previously reported 18. In brief, the mice were anaesthetised with pentobarbital sodium, and then each side of the uterus was infused equal amounts of LPS (1 mg/kg) to induce endometritis. The control group received equal volumes of sterile PBS. To transiently up-regulate the miR-92b expression *in vivo*, the mice were intraperitoneally injected with synthetic agomir-92b (0.5 µmol/kg) for three consecutive days before LPS challenge. The mice were humanely killed at 24 h after LPS challenge to collect uterine tissues for further analysis. All animal experiments were repeated three times.

### qPCR and Western blot

qPCR and Western blot were performed as previously reported 19. qPCR was carried out using the Hairpin-it^TM^ microRNA qPCR Quantitation Kit (GenePharma, Shanghai, China) and LightCycler 96 (Roche, Germany). U6 snRNA was used as an internal control for miR-92b, and the mRNA levels of PTEN, TNF-α and IL-6 were normalized to GAPDH. The 2^-ΔΔCt^ method was employed to calculate relative expression of each gene. For Western blot analysis, the protein blots were incubated with following antibodies: rabbit anti-TLR4 (1:1000, 7074, Cell Signaling Technology, USA), rabbit anti-phospho-NF-κB p65 (1:1000, 3033, Cell Signaling Technology, USA), rabbit anti-NF-κB p65 (1:1000, 4764, Cell Signaling Technology, USA), rabbit anti-β-actin (1:1000, 4970, Cell Signaling Technology, USA), rabbit anti-Bax (1:200, bs-0127R, Bioss, China), rabbit anti-Bcl-2 (1:200, bs-4563R, Bioss, China), rabbit anti-phospho-PI3K (1:200, bs-3332R, Bioss, China), rabbit anti-PI3K (1:1000, ab227204, Abcam, USA), rabbit anti-phospho-AKT (1:1000, ab8932, Abcam, USA), rabbit anti-AKT (1:500, GB111114, Servicebio, China), rabbit anti-β-catenin (1:5000, ab196204, Abcam, USA). The optical density (OD) value of each protein band was quantified using the Image-Pro Plus 6.0 (IPP 6.0) software (Media Cybernetics Inc., USA) and normalized to β-actin.

### Histological Analysis

Uterine tissue samples were fixed in 4% paraformaldehyde overnight, embedded in paraffin, and then sectioned for staining with hematoxylin and eosin (H&E). Finally, the histological changes were observed under a light microscope (Olympus, Japan).

### Immunofluorescence

Cells were seeded in six-well chamber slides to be approximately 30-50% confluent. Cells were washed with phosphate-buffered saline (PBS) and then fixed in 4% paraformaldehyde for 15 min. Cells were permeabilized with 0.2% Triton X-100 on ice for 15 min, washed with PBS, and then blocked with 5% bovine serum albumin (BSA) for 30 min, after which they were incubated with primary antibodies at 4 °C overnight. Cells were then incubated with fluorescein isothiocyanate (FITC)-conjugated secondary antibodies for 2 h at room temperature. And then, nuclei were stained with DAPI for 10 min, and fluorescent images were taken with a fluorescence microscope (Olympus, Japan).

### Immunohistochemistry

Uterine tissue specimens were fixed in 4% paraformaldehyde, embedded in paraffin, and sectioned for incubating with anti-PTEN antibody at 4 °C overnight. And then, the sections were incubated with a horseradish peroxidase (HRP)-conjugated secondary antibody, followed by staining with diaminobenzidine. The sections were washed with PBS and used for microscopic imaging.

### Flow cytometry

The effects of miR-92b on cell apoptosis and cell cycle were detected by flow cytometry. For cell apoptosis analysis, more than 10000 cells of each sample were harvested by trypsin digestion and labeled with Annexin V-FITC and propidium iodide (PI) reagents for 15 min at room temperature in darkness. For cell cycle analysis, more than 10000 cells of each sample were collected and fixed in ethanol at -20 °C. Then, the cells were rehydrated and stained in PI. The stained cells were examined using a flow cytometer (BD Biosciences, USA) and analyzed with FlowJo software (Tree Star, USA).

### Enzyme-Linked Immunosorbent Assay (ELISA)

Uterine tssues were harvested and homogenized with RIPA lysis buffer, and the supernatants were collected after centrifugation at 14,000×g, 4 °C for 15 min. The content of TNF-α and IL-6 in the supernatants was determined with ELISA kits (Biolegend, USA) according to the manufacturer's protocols.

### Statistical analysis

Statistical analyses were conducted using GraphPad Prism 6.0 software (San Diego, CA). Differences between two groups was examined using a Student's *t*-test, and one-way analysis of variance (ANOVA) was used for multiple comparisons. All data are presented as the mean ± standard error of the mean (SEM). A value of *p* < 0.05 was considered statistically significant.

## Results

### Decreased miR-92b expression correlates with cow endometritis

**Figure 1:**
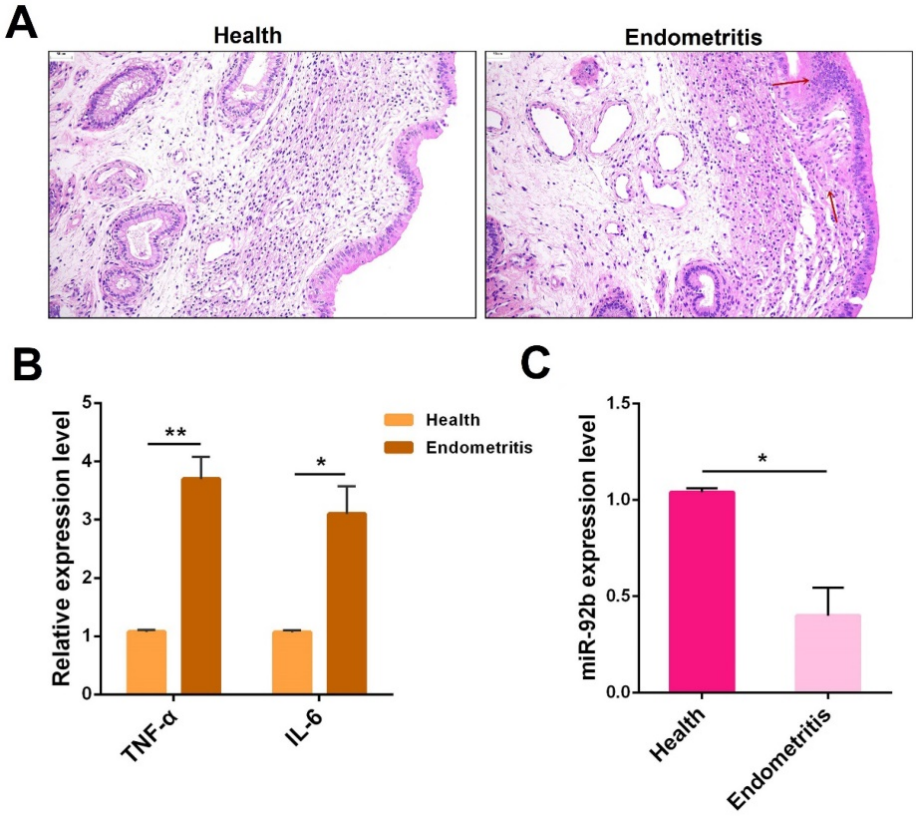
** Expression of miR-92b in endometritis. (A)** Histopathological analysis of uterine tissues (scale bar=50 µm). **(B)** The expression of pro-inflammatory cytokines TNF-α and IL-6 was detected by qPCR. **(C)** The miR-92b expression in uterine tissues was measured by qPCR. Data are expressed as mean ± SEM of three independent experiments. *p < 0.05, **p < 0.01.

Based on a previous miRNA sequencing data showing a reduced expression pattern of miR-92b in endometritis 14, we further validated its expression in cows with endometritis. First, uteri from healthy or endometritis cows were collected and then subjected to H&E staining. Compared with the healthy group, the uteri in the endometritis group exhibited severe pathological changes, characterized by massive inflammatory cell infiltration, epithelial cell detachment, and tissue edema **(Figure 1A)**. Besides, qPCR was employed to determine the expression of the pro-inflammatory cytokines TNF-α and IL-6. And the experimental data showed that these pro-inflammatory cytokines were dramatically increased in inflamed uteri **(Figure 1B)**. Next, the miR-92b expression was also measured by qPCR, and the results indicated that the level of miR-92b in endometritis specimens was lower than that in healthy cows **(Figure 1C)**. These results suggest that down-regulatiton of miR-92b is tightly related to cow endometritis.

### Reduced expression of miR-92b following stimulation with LPS from *E. coli*

**Figure 2:**
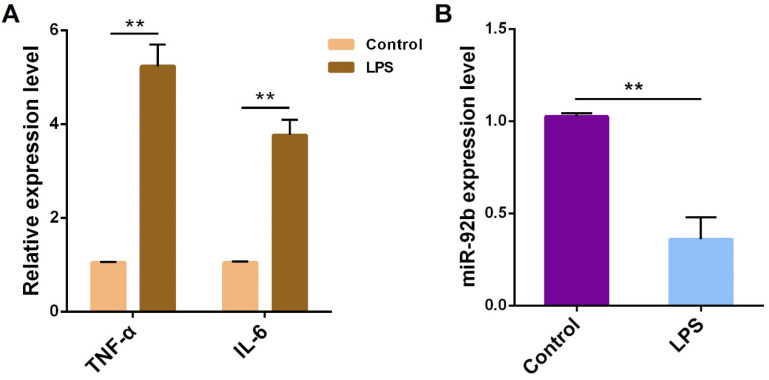
** LPS reduces miR-92b expression in BEND cells.** BEND cells were treated with LPS (10 µg/mL) for 24 h, and the pro-inflammatory cytokines TNF-α and IL-6** (A),** and miR-92b **(B)** were determined by qPCR. Data are expressed as mean ± SEM of three independent experiments. *p < 0.05, **p < 0.01.

To figure out the potential role of miR-92b in the pathogenesis of endometritis, bovine endometrial epithelial cells (BEND cells) were stimulated with 10 μg/mL LPS for 24 h. The expression of TNF-α and IL-6 was obviously increased **(Figure 2A)**, accompanied by a decrease in the expression of miR-92b **(Figure 2B)**. These results further confirm that miR-92b is involved in LPS-mediated inflammatory response.

### miR-92b blunts cellular inflammatory and apoptotic responses

**Figure 3:**
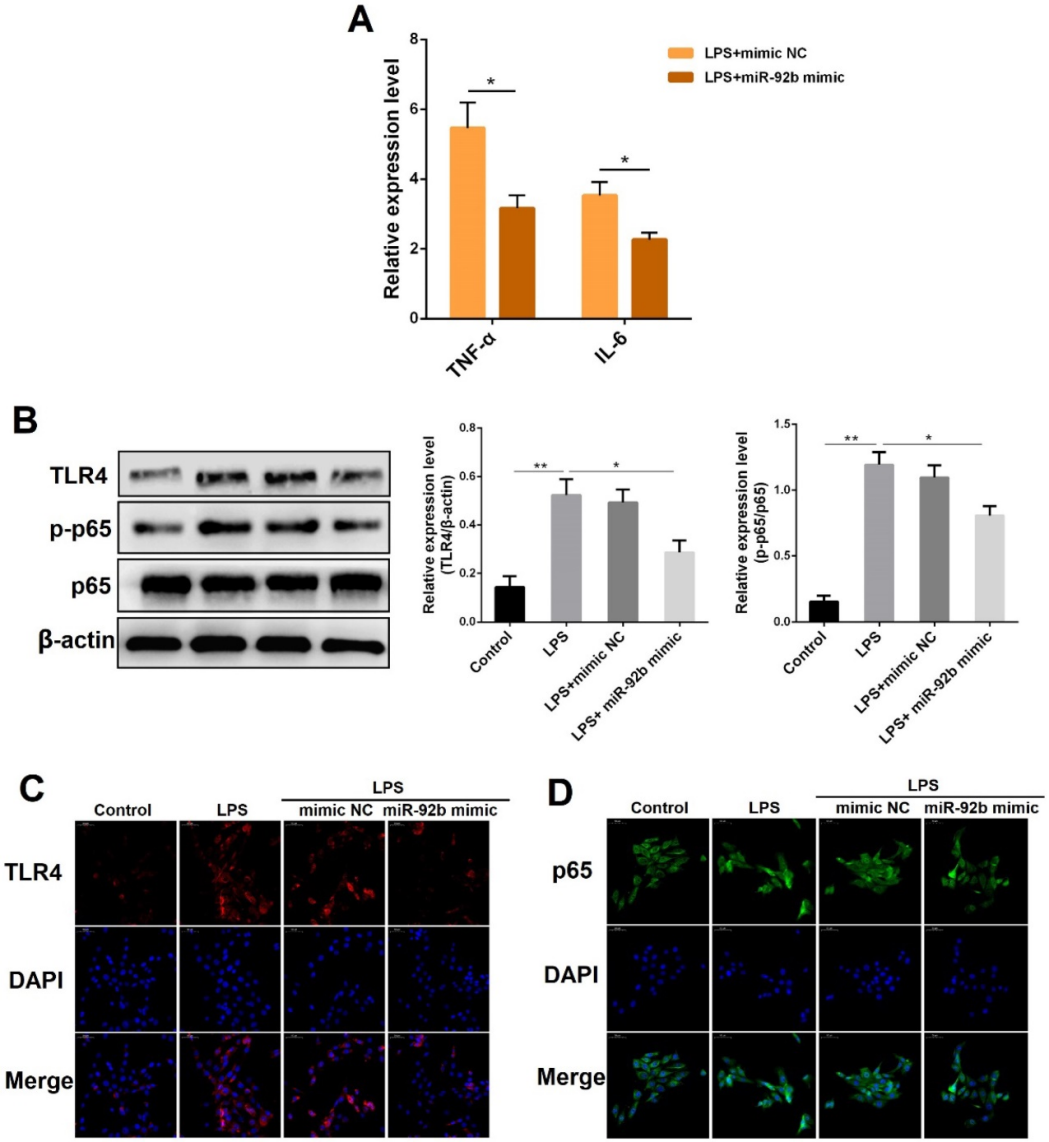
** Overexpression of miR-92b inhibits LPS-triggered inflammation response.** BEND cells were transfected with miR-92b mimic for 24 h, and then treated with 10 µg/mL LPS for another 24 h. **(A)** The expression of IL-6 and TNF-α was detected by qPCR. **(B)** The protein levels of TLR4, p-p65 and p65 were detected by Western blot. **(C)** The expression of TLR4 was further evaluated by immunofluorescence staining (scale bar=50 µm). **(D)** Nuclear translocation of NF-κB p65 was analyzed by immunofluorescence staining. Data are expressed as mean ± SEM of three independent experiments. *p < 0.05, **p < 0.01.

miR-92b is closely associated with tumor development, and has been demonstrated to promote proliferation, migration and invasion of gastric cancer cells by targeting Homeobox D10 20. However, it is unclear whether miR-92b can play a role in LPS-triggered inflammatory responses. BEND cells were transfected with miR-92b mimic for 24 h, followed by 24 h of exposure to LPS, and then the expression of IL-1β and TNF-α was analyzed by qPCR. As displayed in **Figure 3A**, overexpression of miR-92b using miR-92b mimic significantly reduced the production of IL-1β and TNF-α induced by LPS. Furthermore, Western blot results also showed that the protein levels of TLR4 and p-p65 that were up-regulated by LPS were markedly suppressed by overexpression of miR-92b **(Figure 3B)**. In addition, the inhibitory effects of miR-92b on TLR4 and p65 were further confirmed by immunofluorescence staining **(Figure 3C and D)**. The above results indicate that miR-92b negatively regulates LPS-induced inflammatory response by inhibiting the activation of TLR4 and its downstream NF-κB p65.

**Figure 4:**
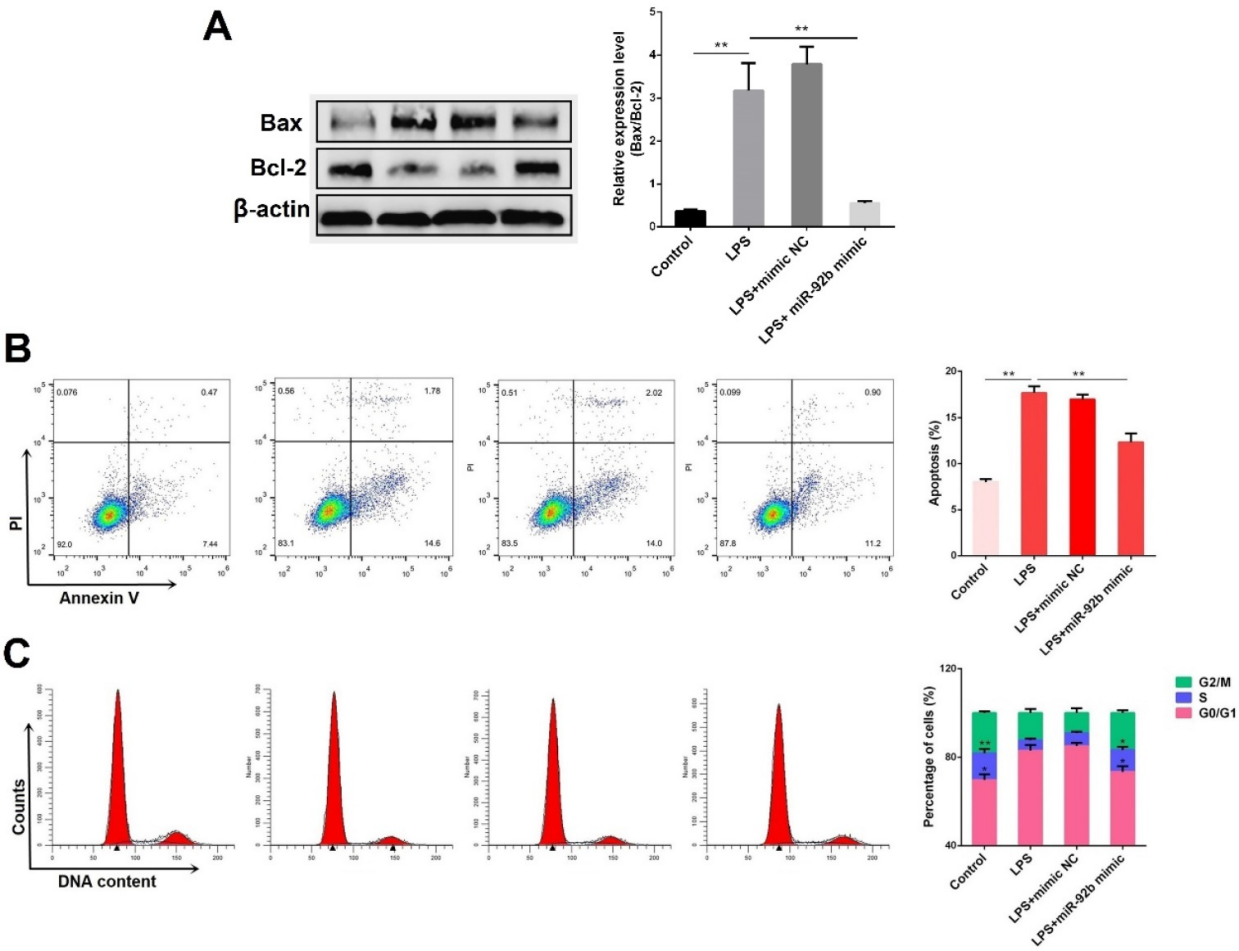
** Overexpression of miR-92b alleviates LPS-induced apoptosis.** BEND cells were transfected with miR-92b mimic for 24 h, and then treated with 10 µg/mL LPS for another 24 h. **(A)** The protein levels of Bax and Bcl-2 were determined by Western blot. **(B)** The cell apoptosis was evaluated by flow cytometry. **(C)** The cell cycle was determined by flow cytometry. Data are expressed as mean ± SEM of three independent experiments. *p < 0.05, **p < 0.01.

Excessive inflammatory response usually induces cell apoptosis 21. The effects of miR-92b on cell apoptosis induced by LPS were subsequently investigated. As shown in **Figure 4A**, the ratio of pro-apoptotic Bax to anti-apoptotic Bcl2 was obviously increased in the LPS-stimulated BEND cells, which was inhibited by overexpression of miR-92b. Besides, the results of flow cytometry analysis showed that overexpression of miR-92b significantly reduced the number of apoptotic cells induced by LPS **(Figure 4B)**. Furthermore, analysis of cell cycle indicated that overexpression of miR-92b also remarkably blocked the inhibitory effects of LPS on cell cycle progression by decreasing G1 period and increasing S period of cells **(Figure 4C)**. The data demonstrate that miR-92b can alleviate LPS-induced apoptosis.

### miR-92b activates the PI3K/AKT/β-catenin pathway

**Figure 5:**
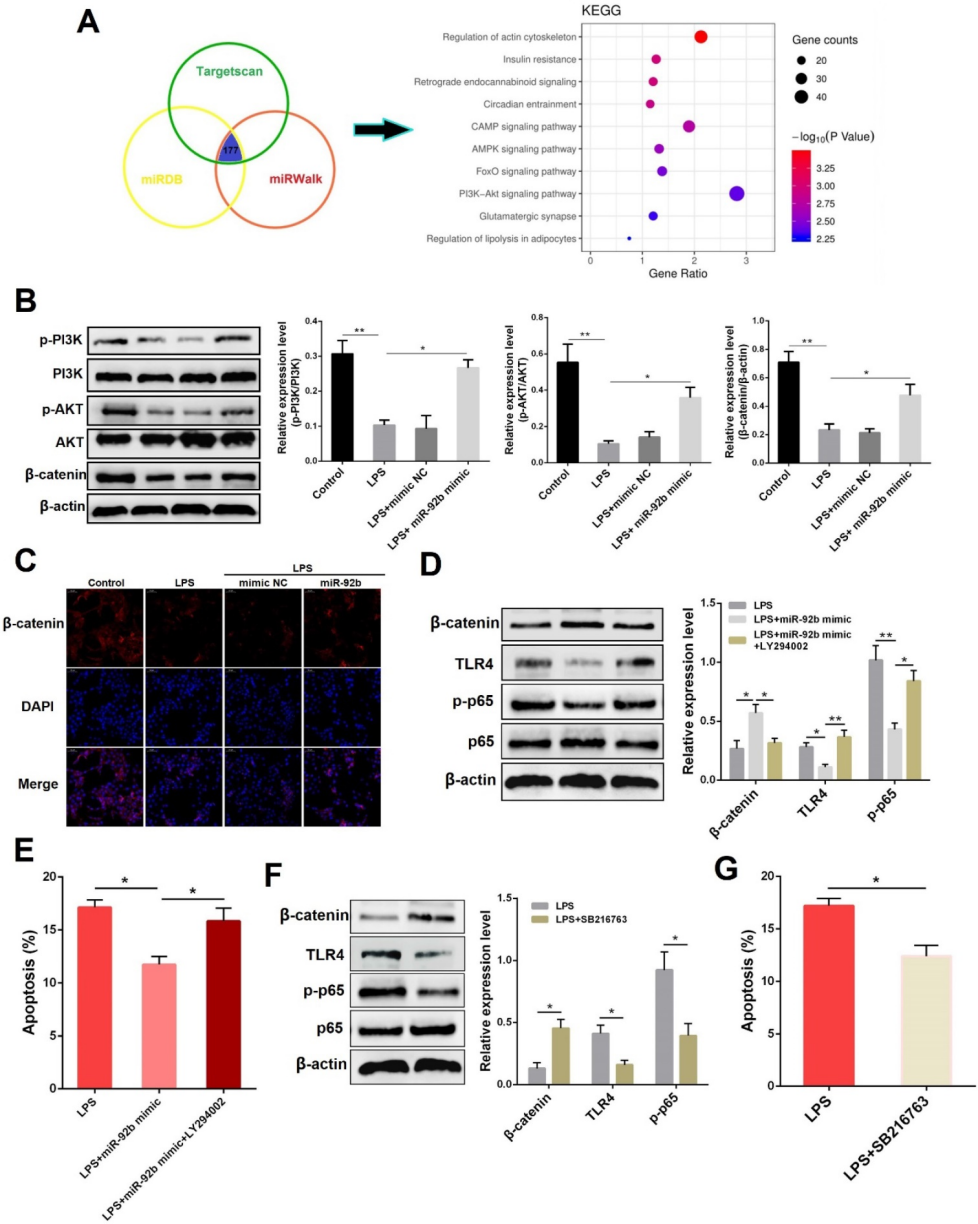
** Overexpression of miR-92b activates the PI3K/AKT/β-catenin pathway. (A)** Enrichment analysis of miR-92b target genes with KEGG. **(B)** BEND cells were transfected with miR-92b mimic for 24 h, followed by 24 h of exposure to LPS (10 µg/ml). The protein levels of p-PI3K, PI3K, p-AKT, AKT and β-catenin in BEND cells were detected by Western blot. **(C)** Cells were treated as (B), and then the cellular location and expression of β-catenin were measured by immunofluorescence staining (scale bar=50 µm). **(D)** BEND cells were transfected with miR-92b mimic for 24 h, and then treated with 10 µg/mL LPS and LY294002 for another 24 h. The protein levels of β-catenin, TLR4 and p-p65 were determined by Western blot. **(E)** Cells were treated as (D), and the apoptosis was detected by flow cytometry. **(F)** BEND cells were pre-treated with SB216763 for 1 h, and then exposed to LPS (10 µg/ml) for 24 h. The protein levels of β-catenin, TLR4 and p-p65 were measured by Western blot. **(G)** Cells were treated as (E), and the apoptosis was analyzed by flow cytometry. Data are expressed as mean ± SEM of three independent experiments. *p < 0.05, **p < 0.01.

To investigate the possible signaling pathways directly targeted by miR-92b, three commonly used miRNA target gene prediction databases (Targetscan, miRDB, and miRWalk) were employed in this study to predict potential target genes of miR-92b. A total of 177 identical target genes were predicted by these three databases, and then the KEGG pathway enrichment analysis was performed on these target genes through the DAVID database. As shown in **Figure 5A**, the target genes of miR-92b were the most enriched in the phosphatidylinositol 3-kinase/protein kinase B (PI3K/AKT) pathway, implying that the biological function of miR-92b is closely related to the PI3K/AKT pathway. PI3K/AKT pathway is tightly associated with cell survival, which can promote cell proliferation and inhibit cell apoptosis 22, and it also plays a regulatory role in inflammation and immune response 23. To confirm the hypothesis, we then analyzed the effects of miR-92b on PI3K/AKT pathway-related proteins (such as PI3K, AKT and β-catenin). Western blot results showed that LPS stimulation significantly inhibited the protein levels of p-PI3K, p-AKT, and β-catenin, whereas overexpression of miR-92b restored the levels of p-PI3K, p-AKT, and β-catenin which were decreased by LPS **(Figure 5B)**. In addition, immunofluorescence staining results also showed that overexpression of miR-92b blocked the degradation of β-catenin promoted by LPS **(Figure 5C)**. The above results reveal that miR-92b positively regulates the PI3K/AKT/β-catenin pathway.

To further determine whether the anti-inflammatory effects of miR-92b were mediated by PI3K/AKT, we used LY294002, a specific inhibitor of PI3K/AKT pathway, and miR-92b to treat BEND cells. As shown in **Figure 5D and E**, inhibition of PI3K/AKT reversed the regulatory effects of miR-92b on β-catenin, TLR4 and p-p65, and also restrained the reduction of apoptotic rate by miR-92b. β-catenin is a co-transcription factor that promotes the transcriptional activation of many target genes after being transferred to the nucleus 24. Intriguingly, overexpression of β-catenin using its agonist SB216763 led to a significant decrease in TLR4 and p-p65 **(Figure 5F)**, as well as apoptotic rate **(Figure 5G)**. Taken together, the above data indicate that miR-92b inhibits TLR4/NF-κB via the activation of PI3K/AKT/β-catenin axis.

### miR-92b down-regulates PTEN expression

**Figure 6:**
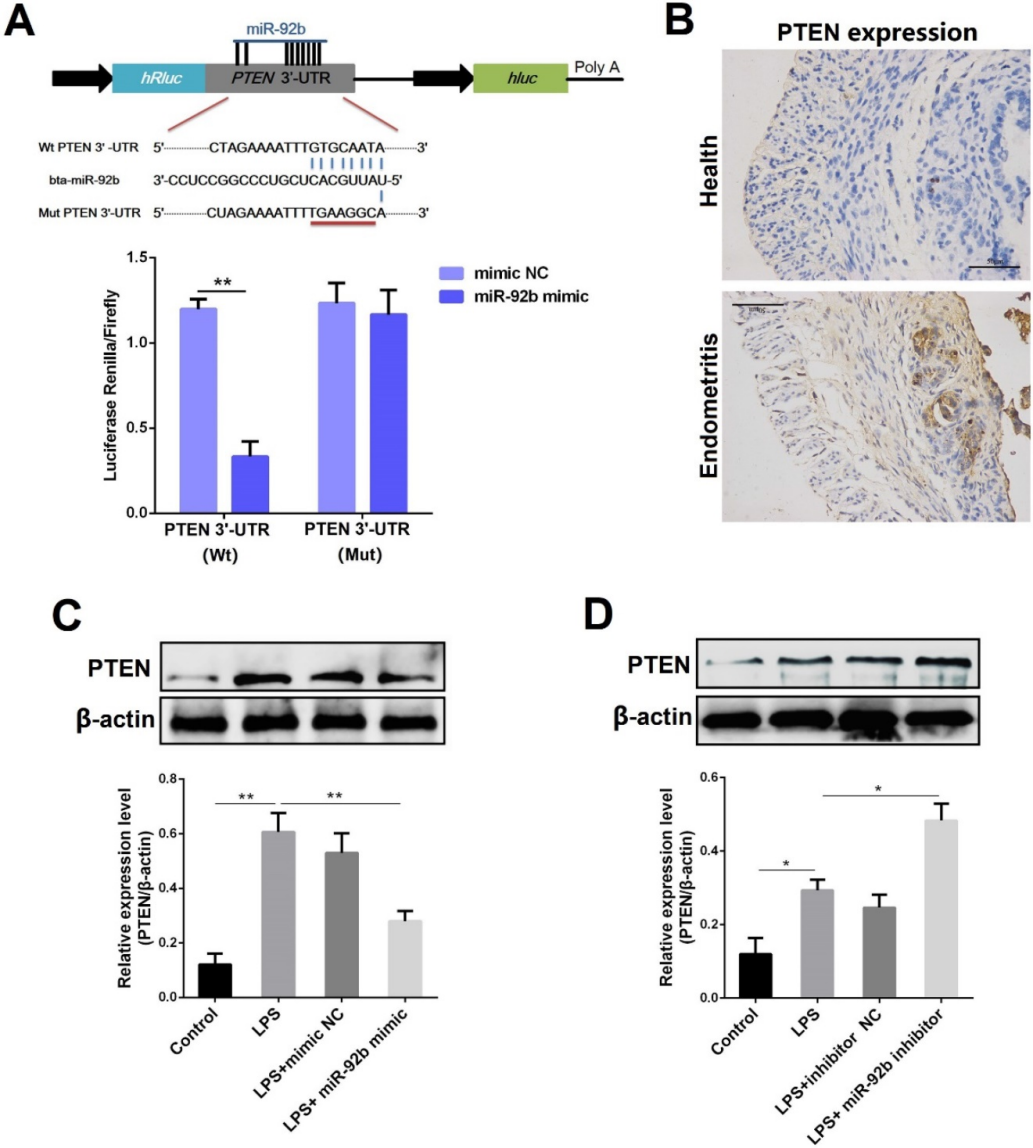
** PTEN is a molecular target of miR-92b. (A)** Double luciferase report assay. **(B)** Immunohistochemical staining of PTEN in the uterine tissues from cow with endometritis (scale bar=50 µm). **(C, D)** BEND cells were transfected with miR-92b mimic or inhibitor for 24 h, followed by 24 h of exposure to LPS. The PTEN expression was detected by Western blot. Data are expressed as mean ± SEM of three independent experiments. *p < 0.05, **p < 0.01.

In general, miRNAs are generally involved in intracellular signaling cascade by affecting the stability of their target genes. 8. Through the prediction of several databases, we observed that there is a putative binding site between miR-92b and the 3′-UTR of PTEN mRNA **(Figure 6A)**. To further confirm the prediction, the luciferase reporter assay was then performed. As displayed in **Figure 6A**, upon co-transfection of 293T cells with miR-92b mimic and wild-type reporter vectors (pischeck-PTEN-wt), the luciferase activity was markedly decreased. However, the inhibition of the luciferase activity by miR-92b was completely abolished when the binding site was mutated. As a natural antagonist of PI3K/AKT, PTEN not only plays an important role in tumor progression, but also mediates inflammation and immune response 25. Interestingly, our results revealed an up-regulated expression level of PTEN during endometritis in dairy cows** (Figure 6B)**. The PTEN protein level was also determined in BEND cells transfected with miR-92b mimic or inhibitor. As shown in **Figure 6C and D**, the expression of PTEN was dramatically decreased by overexpression of miR-92b, whereas inhibition of miR-92b increased PTEN expression. Altogether, these results confirm that PTEN is a molecular target of miR-92b and implicated in the pathogenesis of endometritis.

### PTEN mediates the anti-inflammatory activities of miR-92b

**Figure 7:**
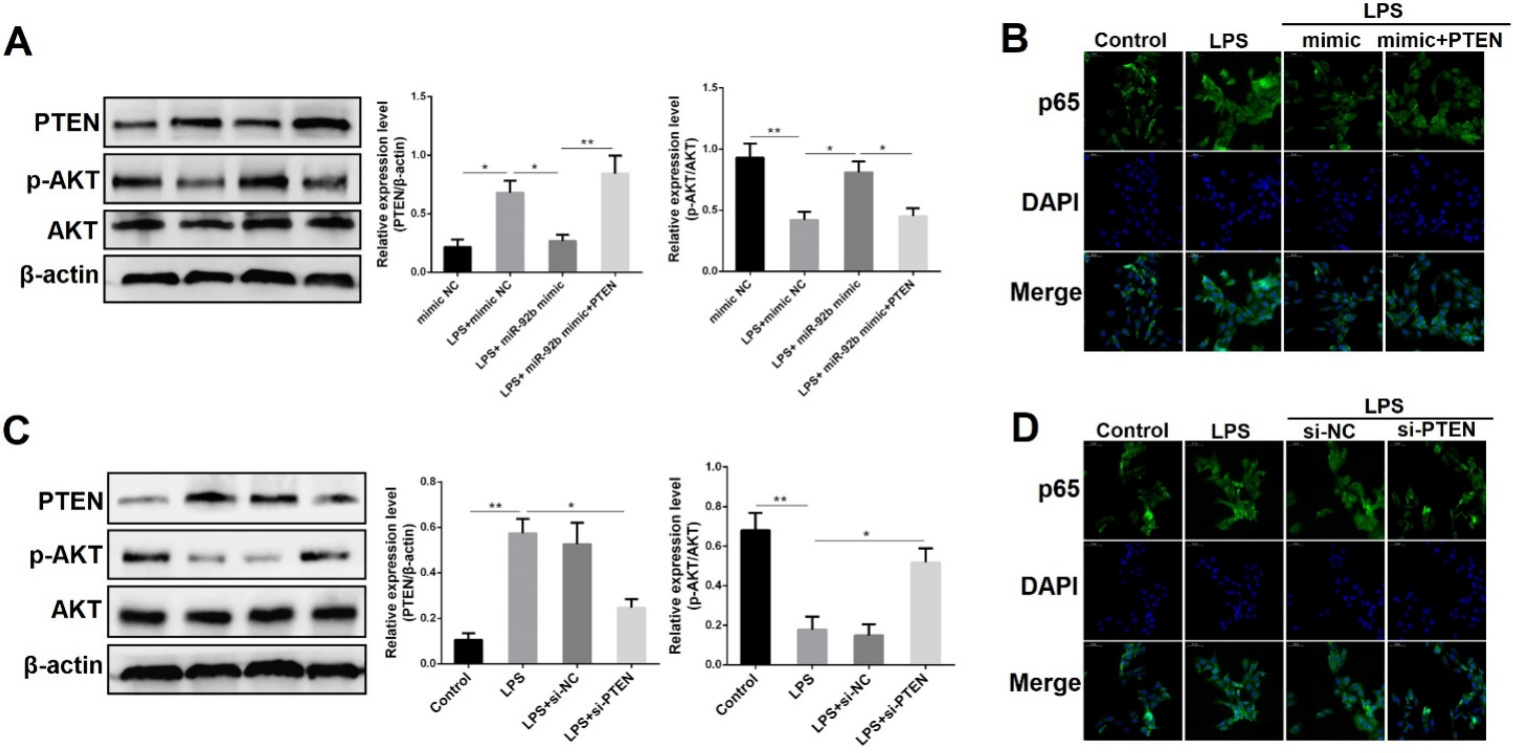
** PTEN mediates the anti-inflammatory effects of miR-92b. (A)** BEND cells were co-transfected with miR-92b mimic and pcDNA3.1-PTEN, and the protein levels of PTEN, p-AKT and AKT were measured by Western blot.** (B)** Cells were treated as (A), and the nuclear translocation of NF-κB p65 was assessed by immunofluorescence staining (scale bar=50 µm). **(C)** BEND cells were transfected with si-PTEN, and the protein levels of PTEN, p-AKT and AKT were measured by Western blot. **(D)** Cells were treated as (C), and the nuclear translocation of NF-κB p65 was analyzed by immunofluorescence staining (scale bar=50 µm). Data are expressed as mean ± SEM of three independent experiments. *p < 0.05, **p < 0.01.

To elucidate whether the anti-inflammatory effects of miR-92b were mediated by PTEN, we examined the protein levels of PTEN and p-AKT following co-transfection with miR-92b mimic and recombinant plasmids overexpressing PTEN (pcDNA3.1-PTEN). As shown in** Figure 7A**, overexpression of miR-92b rescued the LPS-induced down-regulation of p-AKT, while overexpression of PTEN blocked the up-regulation of p-AKT by miR-92b. At the same time, immunofluorescence staining results showed that overexpression of PTEN also hampered the inhibitory effect of miR-92b on the nuclear transcription of NF-κB p65 **(Figure 7B)**.

To further illuminate the key role of PTEN in the inflammatory response induced by LPS, we transfected PTEN siRNA (si-PTEN) into BEND cells to knockdown PTEN expression, and then analyzed the effects of si-PTEN on p-AKT and NF-κB p65. As depicted in **Figure 7C**, knockdown of PTEN not only decreased the expression of PTEN, but also increased the expression of p-AKT. Immunofluorescence staining results further showed that knockdown of PTEN inhibited the nuclear transcription of p65 triggered by LPS **(Figure 7D)**. These data fully validate that PTEN plays a critical role in the inflammatory response of endometrial epithelial cells, and further reveal that miR-92b could attenuate the cellular inflammatory injury by directly targeting the PTEN/PI3K/AKT/β-catenin axis.

### Up-regulation of miR-92b alleviates LPS-induced endometritis in mice

**Figure 8:**
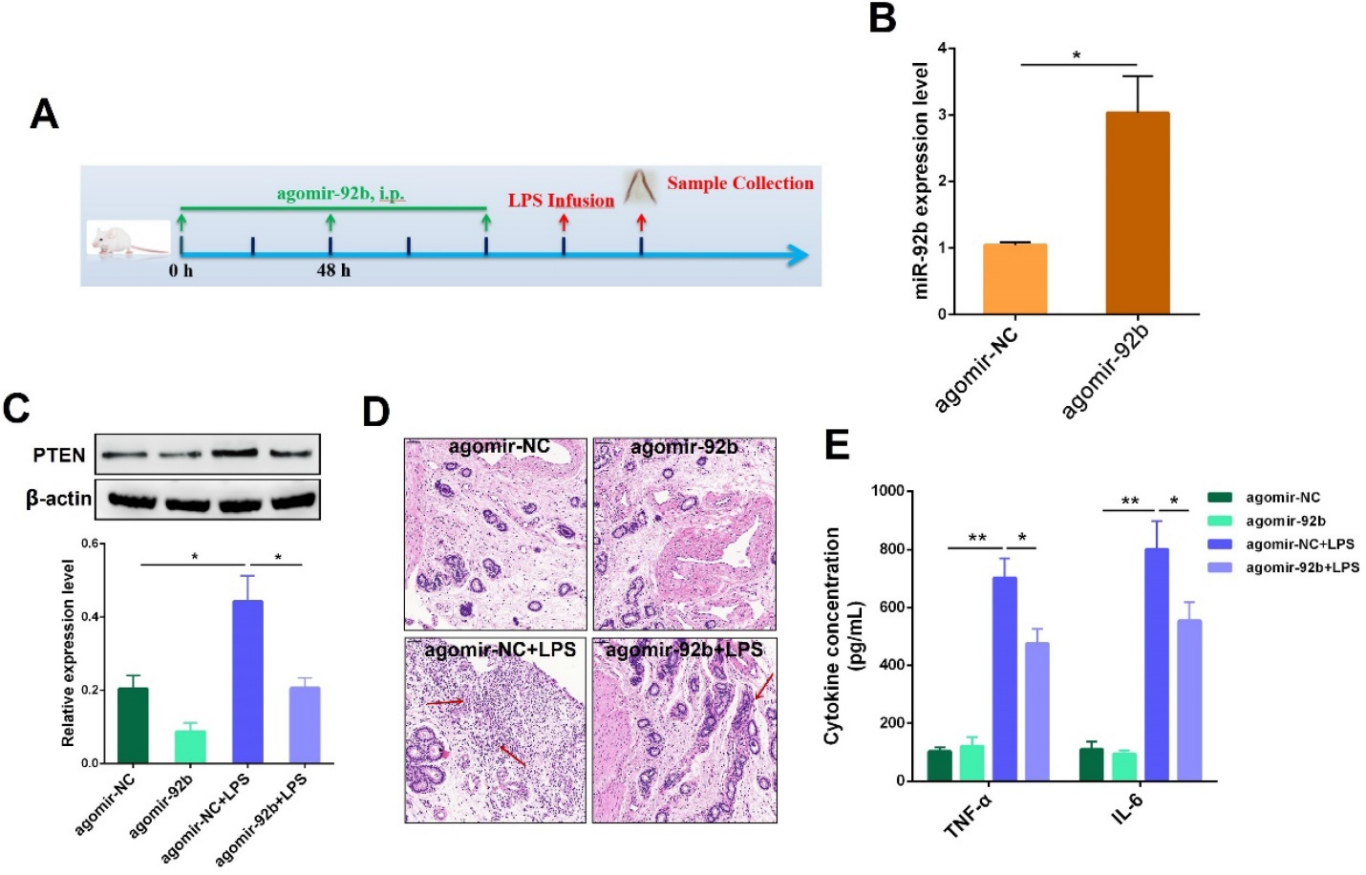
** Delivery of agomir-92b improves LPS-induced endometritis in mice. (A)** A schematic diagram of the experimental design. **(B)** The miR-92b expression was detected in uterine tissues by qPCR. **(C)** The PTEN protein level was measured by Western blot.** (D)** H&E staining of uterine tissues (scale bar=50 µm).** (E)** ELISA measurement of IL-6 and TNF-α in the uterine biopsies. Data are expressed as mean ± SEM of three independent experiments. *p < 0.05, **p < 0.01.

Our *in vitro* studies suggested miR-92b could act as an important negative regulator of PTEN, and thereby mitigate LPS-induced inflammatory injury. Next, to verify the therapeutic potential of miR-92b* in vivo*, agomir-92b was used to transiently increase the miR-92b level in mice, followed by infusion of LPS into the uterine cavity to induce uterine injury **(Figure 8A)**. As shown in **Figure 8B,** treatment with agomir-92b obviously increased the expression of miR-92b in uterine tissues in comparison with agomir-NC. Moreover, a transient increase in miR-92b expression significantly repressed PTEN expression **(Figure 8C)**. Additionally, up-regulation of miR-92b improved the outcome of endometritis, as indicated by the mitigated pathological conditions **(Figure 8D)**, as well as the down-regulation of IL-6 and TNF-α secretion **(Figure 8E)**. Collectively, these results unveil the crucial role of miR-92b in the development of endometritis, and shed new light on the molecular therapy of inflammation-related diseases, such as endometritis.

## Discussion

Cow endometritis caused by various pathogens including Gram-negative and -positive bacteria is believed to affect the reproductive performance of cows by inducing uterine inflammation and damage, which has an adverse impact on the dairy industry 1. Although antimicrobial drugs have been extensively utilized to treat endometritis in dairy cow, the overuse of antimicrobial drugs can lead to problems with drug residues and bacterial resistance, posing a serious challenge to food safety and public health; therefore, it is necessary to study alternative therapeutic strategies for the treatment of endometritis. During bacterial infection of the uterus, the endometrium responds to foreign pathogens by activating or inhibiting specific molecular signals. Thus, a better understanding of these molecular events may contribute to the development of novel molecular diagnostic markers or therapeutic targets for endometritis. In this work, we report for the first time that miR‐92b can act as a protective factor in LPS-induced inflammatory injury by inhibiting the TLR4 signaling via the PTEN/PI3K/AKT/β-catenin axis.

Recently, the role of miRNAs in the inflammatory process has also received growing attention 26, 27. Remarkably, miRNA microarray analysis conducted by Salilew-Wondim et al. identified that miR-92b is closely associated with the development of clinical and subclinical endometritis 14. However, the underlying mechanism by which miR-92b regulates the development of endometritis remains to be further elucidated. Our present study confirmed that the expression of miR-92b was notably decreased during endometritis. Moreover, miR-92b was further observed to be reduced in LPS-stimulated BEND cells, suggesting that miR-92b may function to fine-tune the development of endometritis.

Endometrial epithelial cells express a specific receptor complex that includes TLR4, CD14 and MD2 to recognize endotoxin from pathogenic bacteria 28, 29. In particular, LPS is considered to be one of the major virulence factors of endometrial pathogenic *E. coli* (EnPEC), stimulating a TLR4-dependent inflammatory response in endometrial cells 30, 31. Upon binding to the ligand LPS, TLR4 recruits its downstream molecules, such as MyD88, IRAKs, and TRAF6, which leads to the activation of NF-κB and the occurrence of inflammatory reaction 32. On the other hand, excessive inflammation is thought to induce apoptosis and cause tissue damage 21. Actually, NF-κB activation contributes to cell apoptosis through driving pro-apoptotic p53 signaling 33. Another study directly reported that LPS stimulates apoptosis by TLR4/NF-κB and p53 pathways in intestinal epithelial cells 34. It was found that miR-92b decreased the protein levels of TLR4 and p-p65, as well as the production of pro-inflammatory cytokines. Moreover, miR-92b also inhibited the Bax/Bcl2 ratio and prevented cell apoptosis triggered by LPS. These findings indicate that miR-92b alleviates LPS-induced inflammatory injury, possibly by restraining TLR4-mediated NF-κB activation.

Emerging evidence showed that some miRNAs can act as negative feedback modulators of inflammatory processes through targeting some signaling proteins 13. In the present study, prediction of miR-92b/mRNAs by several databases did not reveal targets in the TLR4 or NF-κB, which indicated that other targets may be involved in the regulation of the TLR4/NF-κB pathway by miR-92b. Intriguingly, KEGG analysis revealed that most of the target genes of miR-92b were enriched in the PI3K/AKT pathway. PI3K/AKT is an important signal pathway, which is closely related to cell apoptosis and inflammation. For instance, PI3K protects neurons from hydrogen peroxide-induced cell death through AKT-mediated anti-inflammatory and anti-apoptotic effects 35. β-catenin is an essential component of the PI3K/AKT pathway, which enters the nucleus to regulate the transcriptional activation of downstream target genes upon activation 23. It has been reported that the activation of β-catenin inhibits cigarette-induced airway inflammation through the PPARδ/p38 pathway 36. Here, we observed that overexpression of miR-92b activated the PI3K/AKT/β-catenin pathway that was suppressed by LPS stimulation, and this effect was significantly reversed after PI3K inhibition. Importantly, PI3K inhibition also blocked the inhibitory effects of miR-92b on the TLR4, NF-κB and cell apoptosis. Indeed, PI3K/AKT has been shown to negatively regulate the NF-κB activation and inhibit myocardial injury in septic septic mice 37. Furthermore, we also found that overexpression of β-catenin dampened the activation of TLR4 and NF-κB and reduced cell apoptosis, illustrating that miR-92b suppresses LPS-induced activation of TLR4/NF-κB via activating the PI3K/AKT/β-catenin pathway.

Next, we further investigated the molecular mechanism through which miR-92b activated the PI3K/AKT/β-catenin pathway. Through the target gene databases and luciferase assay, we verified that PTEN is a molecular target of miR-92b. PTEN is a tumor suppressor with dual-specific phosphatase activity, and can inhibit the phosphorylation of AKT, thereby negatively regulating the PI3K/AKT signaling pathway 38, 39. Studies have also reported that inhibition of PTEN enhances cardioprotection 40, and reduces the degree of brain damage 41. PTEN was observed to be highly expressed during LPS-induced epithelial cell injury, which may explain why PI3K/AKT activity was curbed by high concentration of LPS. PTEN also plays a critical role in inflammation and immune response. For instance, PTEN is implicated in regulating the inflammatory response during the development of bronchial asthma 42. Here, we showed that knockdown of PTEN up-regulated AKT activity and then depressed NF-κB activation. Furthermore, we also demonstrated that PTEN mediated the repressive effects of miR-92b on the inflammatory response induced by LPS. Additionally, PTEN has been revealed to be regulated by β-catenin in ovarian cancer cells 43. Interestingly, our current study also confirmed that β-catenin activation inhibited PTEN expression that was increased by LPS **(Figure S1)**. The result implies that PTEN can not only inhibit the PI3K/AKT/β-catenin pathway, but also is negatively regulated by β-catenin, forming a negative feedback regulatory loop between them. Based on these findings, we conclude that miR-92b activates the PI3K/AKT/β-catenin pathway by directly targeting PTEN, thereby blocking the magnitude of endometrial inflammation.

Animal models of uterine disease have been created in sheep 44 and cattle 45 for the development of new therapeutic strategies, as well as elucidating the immune mechanisms in the uterus. However, the high cost of experimental research in cattle models hindered the studies of cow endometritis to a certain extent. Mice are commonly used as the experimental animal models of various inflammatory diseases, such as mastitis 19 and endometritis 6. Thereafter, to further explore whether miR-92b functions as a potential molecular target for treating endometritis, an experimental murine model of endometritis was generated by intrauterine infusion of LPS. Noteworthily, overexpression of miR-92b using agomiR markedly attenuated the uterine injury, as evidenced by improved pathological conditions. Consistent with *in vitro* results, overexpression of miR-92b also lowered the expression levels of PTEN and pro-inflammatory factors IL-6 and TNF-α.

**Figure 9:**
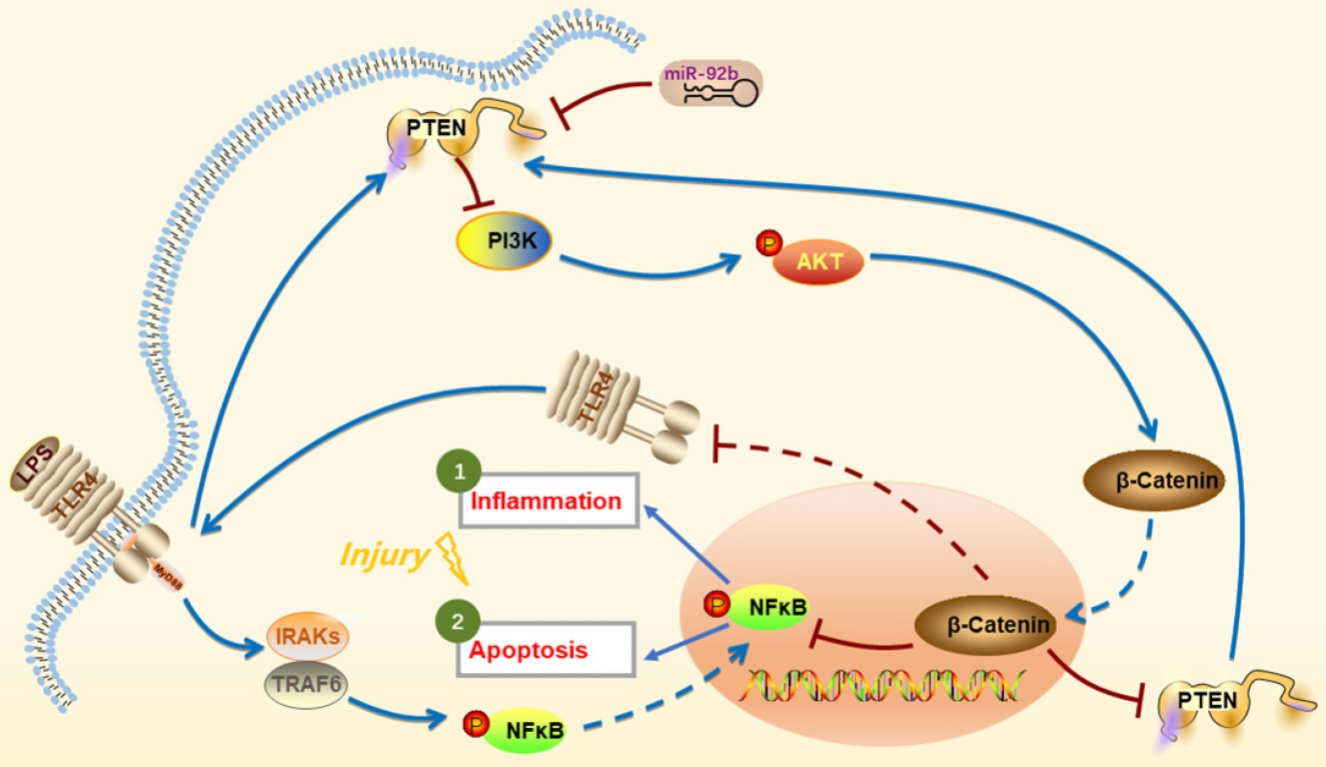
** Schematic diagram of miR-92b action mechanism.** LPS activates IRAKs and TRAF6 through binding to TLR4, leading to NF-κB entering the nucleus and inducing inflammation and cell apoptosis. miR-92b activates the PI3K/AKT/β-catenin pathway by directly targeting PTEN, thereby blocking TLR4/NF-κB-mediated inflammation and apoptosis and then alleviating the inflammatory injury.

In conclusion, we provide the first evidence that up-regulation of miR-92b offers a new intervention to protect against LPS-induced inflammatory injury by down-regulation of PTEN expression and TLR4/NF-κB signaling and restoration of PI3K/AKT/β-catenin signaling **(Figure 9)**. Thus, the study suggests that miR-92b is a potential therapeutic target for endometritis and other inflammatory diseases.

## Supplementary Material

Supplementary figure S1.

## Figures and Tables

**Table 1:** Primer sequence for qPCR

Gene	Primer sequence (5'-3')
TNF-α	F: CTCCTTCCTCCTGGTTGCAG
	R: CACCTGGGGACTGCTCTTC
IL-6	F: CTACCTCCAGAACGAGTATG
	R: CAGCAGGTCAGTGTTTGTGG
GAPDH	F: GGTCACCAGGGCTGCTTT
	R: CTGTGCCGTTGAACTTGC
bta-miR-92b	RT: CTCAACTGGTGTCGTGGAGTCGGCAATTCAGTTGAGGGAGGCCG
	F: GCCGAGTATTGCACTCGTCC
	R: CTCAACTGGTGTCGTGGA
bta-U6	RT: ACGCTTCACGAATTTGCGTGTC
	F: CTCGCTTCGGCAGCACATATACT
	R: ACGCTTCACGAATTTGCGTGTC

## Abbreviations

MiRNAs: MicroRNAs
BEND: Bovine endometrial epithelial
LPS: lipopolysaccharide
TLR4: Toll-like receptor 4
NF-κB: nuclear factor-κB
mRNAs: messenger RNAs
H&E: hematoxylin and eosin
PBS: phosphate-buffered saline
FITC: fluorescein isothiocyanate
HRP: horseradish peroxidase
PI: propidium iodide
ELISA: Enzyme-linked immunosorbent assay
ANOVA: one-way analysis of variance
SEM: standard error of the mean
PTEN: phosphatase and tensin homolog
PI3K: phosphatidylinositol 3-kinase
AKT: protein kinase B
