# Supplementary material for: Enforced expression of miR-92b blunts E. coli lipopolysaccharide-mediated inflammatory injury by activating the PI3K/AKT/β-catenin pathway via targeting PTEN
Source: Int J Biol Sci. 2021 Mar 25;17(5):1289–301. doi: 10.7150/ijbs.56933 (PMC8040465; doi:10.7150/ijbs.56933)
Supplement: Supplementary file 1 — Supplementary figure S1. [file ijbsv17p1289s1.pdf]

Figure S1

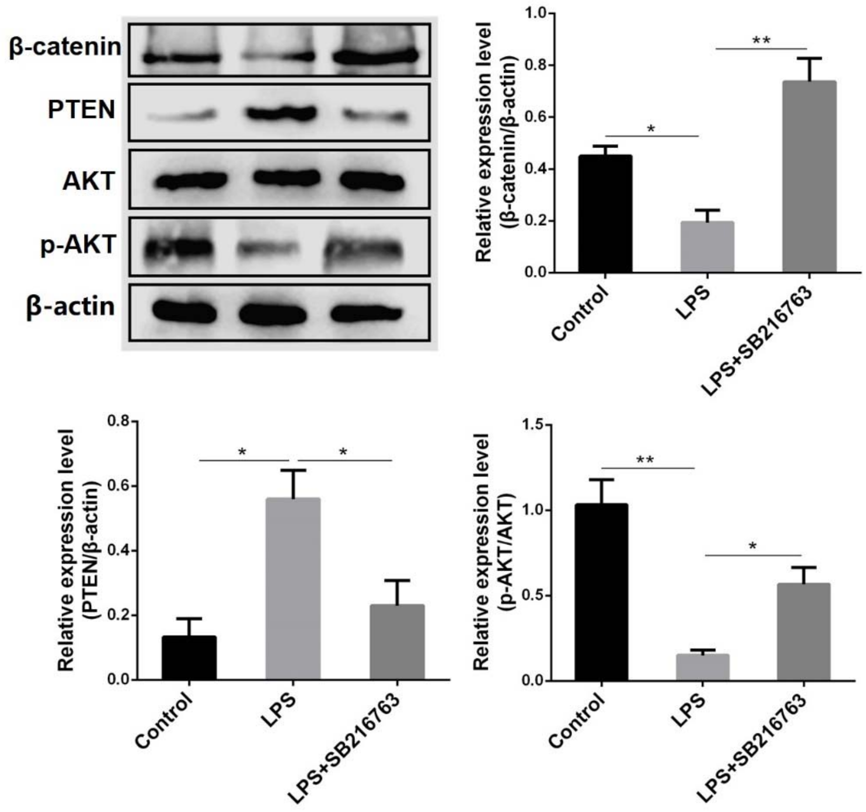

Figure S1. β-catenin inhibits PTEN expression. Western blot analysis of the β-catenin, PTEN, p-AKT and AKT levels in BEND cells treated with SB216763. Data are expressed as mean ± SEM. $^{*}p < 0.05$, $^{**}p < 0.01$.
